# Implementation of guidelines on family involvement for persons with psychotic disorders in community mental health centres (IFIP): protocol for a cluster randomised controlled trial

**DOI:** 10.1186/s12913-020-05792-4

**Published:** 2020-10-09

**Authors:** Lars Hestmark, Maria Romøren, Kristin Sverdvik Heiervang, Bente Weimand, Torleif Ruud, Reidun Norvoll, Kristiane Myckland Hansson, Irene Norheim, Eline Aas, Elisabeth Geke Marjan Landeweer, Reidar Pedersen

**Affiliations:** 1 Centre for Medical Ethics, University of Oslo, Kirkeveien 166 Fredrik Holsts hus, 0450 Oslo, Norway; 2 Division of Mental Health Services, Akershus University Hospital, Sykehusveien 25, 1474 Nordbyhagen, Norway; 3 Faculty of Health Sciences, Oslo Metropolitan University, Oslo, Norway; 4 School of Nursing and Midwifery, Queens University, Belfast, Northern Ireland; 5 Institute of Clinical Medicine, University of Oslo, Oslo, Norway; 6 Work Research Institute, Oslo Metropolitan University, Oslo, Norway; 7 Division of Mental Health and Addiction, Vestre Viken Hospital Trust, Lier, Norway; 8 Department of Health Management and Health Economics, University of Oslo, Oslo, Norway; 9 Department of General Practice and Elderly Care Medicine, University of Groningen, University Medical Center Groningen, Groningen, the Netherlands

**Keywords:** Family intervention, Psychotic disorders, Schizophrenia, Family psychoeducation, Family involvement, Mental health service research, Clinical ethics, Implementation

## Abstract

**Background:**

Family involvement for persons with psychotic disorders is under-implemented in mental health care, despite its firm scientific, economic, legal and moral basis. This appears to be the case in Norway, despite the presence of national guidelines providing both general recommendations on family involvement and support in the health- and care services, and specific guidance on family interventions for patients with psychotic disorders. The aim of this project is to improve mental health services and the psychosocial health of persons with psychotic disorders and their relatives, by implementing selected recommendations from the national guidelines in community mental health centres, and to evaluate this process.

**Methods:**

The trial is cluster randomised, where 14 outpatient clusters from community mental health centres undergo stratified randomisation with an allocation ratio of 1:1. The seven intervention clusters will receive implementation support for 18 months, whereas the control clusters will receive the same support after this implementation period. The intervention consists of: 1. A basic level of family involvement and support. 2. Family psychoeducation in single-family groups. 3. Training and guidance of health care personnel. 4. A family coordinator and 5. Other implementation measures. Fidelity to the intervention will be measured four times in the intervention arm and two times in the control arm, and the differences in fidelity changes between the arms constitute the primary outcomes. In each arm, we aim to include 161 patients with psychotic disorders and their closest relative to fill in questionnaires at inclusion, 6 months and 12 months, measuring psychosocial health and satisfaction with services. Clinicians will contribute clinical data about patients at inclusion and 12 months. Use of health and welfare services and work participation, for both patients and relatives, will be retrieved from national registries. We will also perform qualitative interviews with patients, relatives, health care personnel and leaders. Finally, we will conduct a cost-effectiveness analysis and a political economy analysis.

**Discussion:**

This project, with its multilevel and mixed methods approach, may contribute valuable knowledge to the fields of family involvement, mental health service research and implementation science.

**Trial registration:**

ClinicalTrials.gov Identifier NCT03869177. Registered 11.03.19.

## Background

There are compelling reasons to intensify the implementation of family involvement in mental health care, particularly for persons with severe mental illness. This study limits its scope to psychotic disorders [1], which are characterised by severe, enduring symptoms and functional and social challenges, affecting the psychosocial health, coping abilities and communication patterns of both patients and their families [2, 3].

We intend the terms ‘family’ and ‘relative’ to cover anyone who provides substantial and unpaid support to a person with a psychotic disorder, including friends and other significant persons. The concept ‘family involvement’ comprises both a basic level of involvement and support and family interventions, such as family psychoeducation [4]. The basic level includes meeting the relatives, assessing their strengths, burdens and needs, establishing a system of safety (crisis plan), listening to their experiences, concerns and preferences, receiving their information about the patient and providing them with general information about the health service, the illness and where they can obtain further support [5]. This necessary foundation may also constitute the initial phase of family psychoeducation, where the patient and relatives can develop coping strategies and helpful communication patterns [4].

Research indicates that family interventions may improve social function, self-experienced health and adherence with medication, as well as reduce the frequency of relapse, hospital admissions and days spent in hospital for persons with psychotic disorders [6–10]. Evidence also suggests that such interventions may improve the experience of caregiving, the quality of life among family members and family function, and further reduce the family burden, levels of ‘expressed emotion’ and relatives’ psychological distress [6, 11–15]. Economic analyses, of family-based interventions versus standard care only, consistently report net saving in direct or indirect costs [6]. Family psychoeducation has the most solid evidence-base among these interventions [2] and is highly compatible with other pillars of psychiatric treatment, including antipsychotic medication and cognitive-based therapy. However, various family interventions have several elements in common, even if deriving from contrasting philosophical and therapeutic traditions [16].

We also consider it a moral imperative to involve those providing unpaid care and support, in collaboration with professional care. The deinstitutionalisation of mental health care services in high-income countries has led to an increase in caring responsibilities for relatives, and their efforts are estimated to save the public health services significant costs [11]. Yet, regardless of the documented benefits and a broadly acknowledged ethical and legal rationale, studies indicate that family caregivers for persons with severe mental illness experience less involvement, cooperation and support than they feel is adequate [17]. The poor implementation of family interventions in mental health care points to a similar tendency [18, 19]. This may be due to both specific barriers to implementing family involvement in mental health care, and barriers that are more general to translating evidence-based treatment into everyday clinical practice [18, 20–22].

Health authorities in several countries have attempted to bridge the gap between scientific evidence and clinical practice by launching guidelines that recommend family interventions as a first-line treatment during all stages of psychotic disorders [23–26]. Such clinical guidelines are based on evidence synthesis from individual studies, where skilled and motivated clinicians provide an intervention to study participants, who may be carefully selected through narrow inclusion and exclusion criteria. Yet, to implement these guidelines in everyday practice, non-selected clinicians are supposed to change their clinical practice towards unselected patients and families with various comorbidities. The pathway from evidence generation to evidence synthesis and guideline development is well developed, whereas the pathway from evidence-based guidelines to evidence-based practice has more recently come to attention.

In Norway, the Directorate of Health has launched national guidelines on families/next of kin in the health- and care services. These are general recommendations on family involvement and support based on ethical considerations, legal regulations, research evidence and discussions between key stakeholders and experts [27]. Additionally, the national guidelines on the treatment of psychotic disorders and the newly launched clinical pathways in mental health care specifically recommend family interventions as a first-line treatment of psychotic disorders [28, 29]. Preliminary mapping indicate that the implementation of these guidelines vary considerably in Norwegian community mental health centres (CMHCs). However, we know little about whether implementing the national guidelines in a naturalistic setting would be associated with improved outcomes for patients, relatives and the public health and welfare services.

Within this context, our project group will develop, conduct and evaluate a complex intervention [30] to implement guidelines on family involvement for persons with psychotic disorders in Norwegian CMHCs. Through a pragmatic trial design, we will employ mixed methods to investigate and explore the implementation process in a naturalistic setting. Fidelity scales will be used to assess and influence the implementation, inspired by the groundbreaking work of the US National Evidence-Based Practices (NEBP) project and its Norwegian counterpart ‘Bedre psykosebehandling’ (BPB), both large-scale studies on the implementation of evidence-based practices for persons with psychotic disorders [31, 32]. Our implementation support will target a wide spectrum of clinical outpatient units and their non-selected personnel, while we measure and compare changes in implementation-, service- and client outcomes, between intervention and control sites. To study this particular intervention, a cluster-randomised design is appropriate and necessary to minimise contamination.

### Objectives

#### Primary objective

- To evaluate whether our implementation support is associated with a higher level of implementation of the selected recommendations in the national guidelines.

#### Secondary objectives

- To measure the current level of implementation of the selected recommendations in the national guidelines in participating clinical units.
- To explore barriers to and facilitators for implementing the national guidelines among the stakeholders at the clinical, organisational, and policy level.
- To explore moral dilemmas and conflicting interests related to family involvement, and strategies on how to resolve them.
- To investigate whether a higher level of implementation of the selected recommendations is associated with improved outcomes for patients and relatives.
- To analyse whether outcomes for patients, relatives and the public health and welfare services, justify the costs of implementing family involvement for persons with psychotic disorders.

### Trial design

**Fig. 1:**
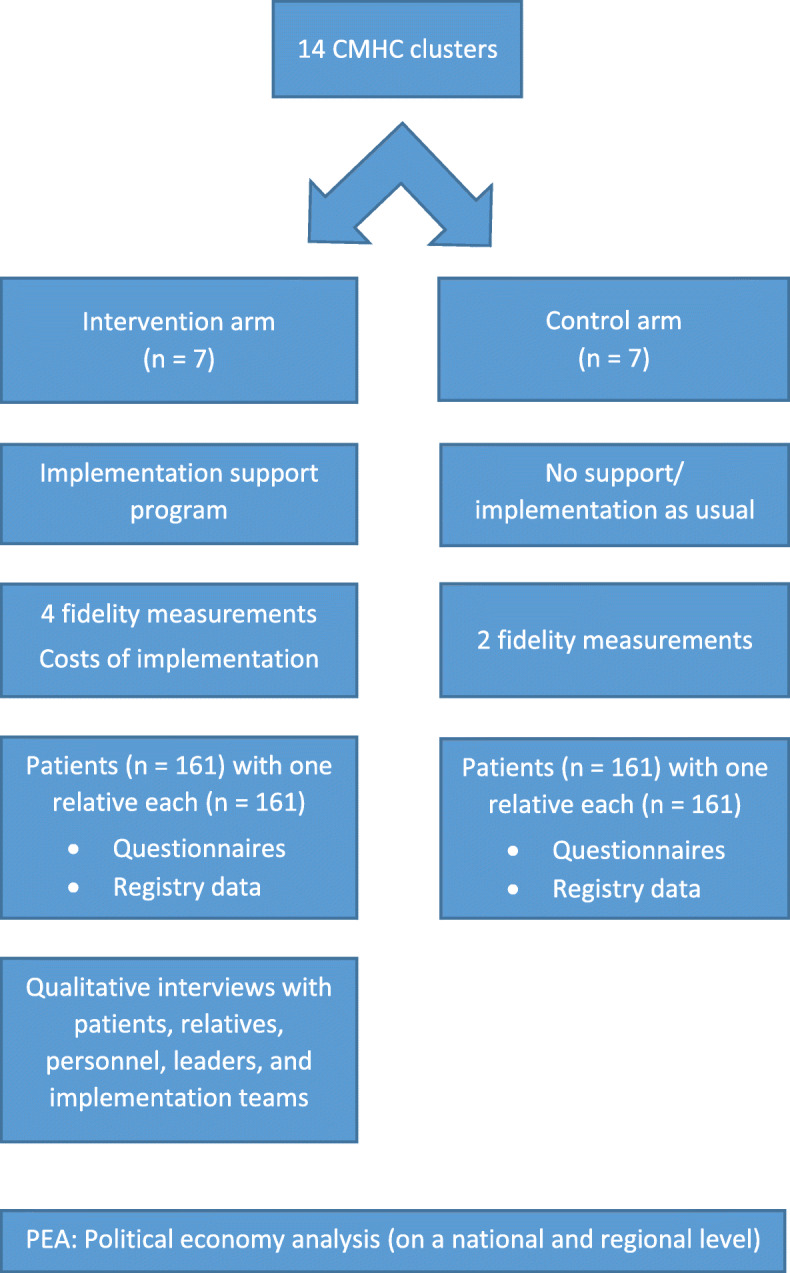
The study design of the IFIP trial

The study is a cluster randomised controlled trial, employing stratified randomisation with an allocation ratio of 1:1 within each block. The clinical outpatient unit(s) with the main responsibility of treating patients with psychotic disorders, in their discrete geographical catchment area, will constitute a single cluster and unit of randomisation. Please see Fig. 1 for a general overview of the study design. This article conforms to the Standard Protocol Items: Recommendations for Interventional Trials (SPIRIT) [33].

## Methods

### Setting

We selected five counties in the South-Eastern Norway Regional Health Authority to limit travel distances and the use of project resources. The selected counties had 16 CMHCs (In Norwegian ‘Distriktspsykiatrisk Senter (DPS)’), which were composed of both inpatient and outpatient units. Of these 16 centres, 12 agreed to participate in the study. The main reason given for non-participation was the lack of capacity to engage in a research project. Preliminary mapping indicate major differences, both between and within centres, in the level and character of family involvement. Furthermore, the distinct populations covered by the various centres show substantial differences in size, ethnic composition and median income level. A comprehensive list of study sites is available at clinicaltrials.gov.

### Selection, sample size and allocation of clusters

To be eligible as clusters, clinical outpatient units had to be part of a participating CMHC and have the main responsibility of treating patients with psychotic disorders in their discrete geographical catchment area. We accepted all types of clinical outpatient units, from Flexible Assertive Community Treatment teams (FACT) [34] to stationary outpatient clinics. The study recruited both clinics solely dedicated to the treatment of psychotic disorders and units covering a wider spectrum of conditions, including substance abuse and bipolar disorder. Some of the centres had multiple outpatient units caring for patients with psychotic disorders. When these covered the same area, we invited all of them to participate in the same cluster. After having recruited 15 clinical units in total, the project had to join two of them in order to have 14 clusters for randomisation. We merged the two units who collaborated the most into a single cluster, and these came from different CMHCs. In line with the pragmatic nature of the trial, there were no exclusion criteria for clusters.

Based on the average results from the NEBP project [31] and similar research we used a mean difference in fidelity scores of 1.82 with an average standard deviation (SD) of 0.80, after 18 months of implementation support to calculate sample size [35, 36]. Choosing 5% two-tailed significance and 80% power, we estimated that 4 clusters in each arm were needed to show that implementation support gives a significant increase in fidelity, compared to baseline or low fidelity. Since these previous studies were not randomised, a premise for this calculation is that the mean fidelity will not change in the control arm. To secure sufficient power in the quantitative study on patients and relatives (see below), we recruited 7 clusters to each arm.

The project group generated a sequence by ranking the clusters from 1 to 14, according to their current number of patients with psychotic disorders. We then stratified the clusters into three blocks; 4 clusters with between 130 and 217 patients, 6 clusters with between 60 and 129 patients, and 4 clusters with between 1 and 59 patients. Within each block, the clusters were randomised to either the intervention or the control arm, with an allocation ratio of 1:1. An independent statistician performed the allocation, drawing 14 numbers using the Microsoft Excel RAND-function, being blind to both the sequence of clinical units and the stratifying variable. The purpose of doing stratified randomisation was primarily to achieve a balance in the number of patients and relatives between the two arms, and secondly to include units of various sizes in both. Since the larger units are located in metropolitan areas, the stratification inadvertently resulted in both urban and rural clusters in each arm.

### Interventions

The clinical units in the intervention arm will receive implementation support for 18 months to assist the implementation of selected recommendations in the national guidelines. The control units will receive training and guidance only after this period. Meanwhile, control sites will not be obligated to follow any specific practice. Since the IFIP intervention is a complex intervention, this section is structured after the Medical Research Council (MRC)‘s framework to give a clear overview [30]. The framework was used actively to guide the development- and feasibility-stages described below.

### Development

In the development phase, our project group selected recommendations in the national guidelines on family involvement for persons with psychotic disorders based on the following non-ranked criteria: a) scientific evidence of relevant and favourable outcomes for patients, relatives, or the public health and welfare services; b) legal regulations and requirements; c) feasibility for the mental health services; and d) acceptability and relevance to patients, relatives and clinicians. We developed the IFIP intervention in conjunction with the selection of appropriate outcome measures in an interactive process to cluster the selected recommendations into key interventions [37].

Inspired by a responsive evaluation approach [38], the project group carried out an assessment by panel groups of 3–9 participants, one for each of the three main stakeholders; i.e. patients, relatives, and clinicians. Through these, we explored the acceptability, feasibility and relevance to the main stakeholders of the selected recommendations, the key interventions and the proposed outcome measures. We also appointed a stakeholder committee to give advice throughout the project. The members of this committee, and representatives of the cooperating CMHCs, were given the opportunity to review the same elements. Based on this preliminary exploration, we made significant changes both to the contents of the IFIP intervention and the outcome measures, before the start of data collection and implementation. For instance, relatives emphasised the need to speak to the patient’s primary clinician, and not just the family coordinator, to be involved in treatment decisions. The intervention therefore includes at least one meeting between the primary clinician, patient and relative(s). Family workers were concerned that family involvement would remain their exclusive domain and not be adopted by all clinicians as a standard approach. Thus, the intervention and implementation strategy employs a whole-ward approach, where all clinicians will be offered training in basic family involvement and FPE. A few psychometric instruments in the questionnaires were substituted by other measures because the respondents found them stigmatising and/or not accurate in addressing their situation.

The resulting IFIP intervention consists of the following elements (see Additional file 1):

I. Clinical interventions
  1.1 A basic level of family involvement and support
  1.2 Family psychoeducation in single-family groups
II. Implementation interventions
  2.1 Training and guidance of health care personnel
  2.2 A family coordinator
  2.3 Other implementation measures

### Piloting, feasibility, evaluation and reporting

Recently the Norwegian research project BPB conducted and evaluated a large-scale implementation of family psychoeducation, among other evidence-based practices for persons with psychotic disorders, employing fidelity scales and questionnaire-based outcomes [32]. Clinical, procedural and methodological input from that project limits our need for a full-scale pilot with correspondent evaluation and reporting, beyond the feasibility and acceptability assessments outlined above. The basic level of family involvement and support has not been tested and evaluated in a similarly rigorous way. However, we consider this element a necessary foundation for family psychoeducation and a similar model was piloted with limited, but positive, evaluation [39].

### The implementation strategy

The implementation strategy will be adapted continuously in response to local requirements and conditions, as well as data and feedback from the clinical units. A comprehensive and final account of this process will therefore be available only after the implementation period is finished. Our approach is based on the groundbreaking work of the NEBP and its Norwegian counterpart BPB, adapting relevant strategies, tools and fidelity scales from these projects to suit the IFIP trial. The central components of our implementation strategy are listed as ‘implementation interventions’ in the IFIP intervention (2.1–2.3). Training and guidance of health care personnel and the appointment of a family coordinator are both part of the strategy to implement the clinical interventions. At the same time, the national guidelines recommend these two elements as permanent organisational structures which themselves need to be implemented. Thus, we will encourage the services to gradually assume responsibility for these elements and implement them on a permanent basis. Element 2.3 lists the remaining components of our implementation strategy, to support the implementation of both the clinical interventions and the permanent implementation interventions. The components include a focus on management commitment and support, a local implementation team, kick-off sessions, fidelity assessments with systematic feedback, work plans, network meetings, and exchange of experiences and tools (see Additional file 1). Our implementation strategy addresses all of the five major domains in the Consolidated Framework for Implementation Research (CFIR): characteristics of the program (e.g., evidence strength and quality, complexity); the outer setting (e.g., patient/relatives’ needs and resources); inner setting (e.g., compatibility of the intervention with existing programs, leadership engagement); the process used to implement the program (e.g., quality and extent of planning, engagement of key stakeholders) and characteristics of individuals involved (e.g., knowledge and attitudes) [40], although we did not use this framework actively when designing the strategy.

### Participants

The IFIP trial has three categories of participants: Patients, relatives, and clinicians. These will be recruited from the participating clusters to take part in the quantitative and qualitative studies described later in this article. A political economy analysis will involve further stakeholders, as detailed later.

### Clinicians

Clinicians in the participating units perform a wide range of tasks in this trial. They will recruit patients and relatives, collect clinical data, and measure selected clinical outcomes. In addition, they will participate in research themselves, by taking part in fidelity assessments and qualitative interviews, or answering questionnaires. Apart from these mainly research-related activities, the clinicians in the intervention arm will also help implement and provide better family involvement for patients with psychotic disorders. There are no baseline requirements of the local staff, such as specific training, competency or professional background.

### Patients and relatives

Patients and relatives will be included in dyadic pairs by the local clinicians.

#### Patients’ inclusion criteria

- To have an established psychotic disorder (F20–29) [1] or a tentative diagnosis of psychotic disorder, certain enough to begin treatment. This need not be the patient’s primary diagnosis. Clinicians do not have to use a specified instrument or procedure to diagnose the patient, but must record how the diagnosis was made.
- To be 18 years or older at the time of inclusion.

#### Patients’ exclusion criteria

- To be sentenced to psychiatric treatment (forensic clients).
- Not being competent to consent to participation in research.
- Having completed more than five joint sessions of family psychoeducation in single-family groups (patient and relative together) or more than ten joint sessions (multiple families together) in multiple-family groups, or a similarly structured family intervention. Does not apply to participants in the qualitative studies.
- Not having any relatives or next of kin (see definition below).

#### Relatives’ inclusion criteria

- Being a relative of a patient with a diagnosis as described above. We use the term ‘relative’ broadly, to signify any family member, close friend, next of kin, or other significant person who support the patient, without being a professional/paid helper.
- To be 18 years or older at the time of inclusion.

#### Relatives’ exclusion criterion

- Having completed more than five joint sessions (patient and relative together) of family psychoeducation in single-family groups or more than ten joint sessions (multiple families together) in multiple-family groups, or a similarly structured family intervention. Does not apply to participants in the qualitative studies.

Patients and relatives must fulfill the criteria above and patients must receive treatment in a participating clinical unit at inclusion, but there are no further requirements. Recruitment of these pairs should be entirely independent from the decision to offer family involvement and other treatment. This means that recruited patients and relatives do not have to receive any specific treatment, intervention, or support during the trial period, in neither the intervention nor the control arm. Correspondingly, the patients and relatives receiving a project-backed intervention, such as family psychoeducation, do not have to participate in the study. For example, forensic clients and their relatives can benefit from family involvement, without taking part in the research. Disconnecting research from treatment in this way serves an ethical purpose, by not favoring study participants with better care. However, there is also an academic rationale: to investigate the impact of improved family involvement practices in the clinical unit on a wider group of patients and relatives, and not just those who received a particular intervention.

### Outcomes

Evaluations of complex interventions usually require a complementary use of quantitative and qualitative methods, to investigate and inform the process [41]. Following Proctor et al.’s framework for implementation research outcomes, our study comprises implementation outcomes (acceptability, adoption, appropriateness, fidelity, penetration, and costs), service outcomes (efficiency, effectiveness, and patient-centeredness) and client outcomes (satisfaction, function, and symptomatology) [42].

### Intervention fidelity

In this part, we seek to quantify the implementation of the selected national guidelines by employing three five-point fidelity scales, where 1 equals poor fidelity and 5 equals high fidelity. Researchers use such scales to assess and influence the implementation process based on the hypothesis that the replication of core elements, previously tested through rigorous research designs, will achieve similar outcomes [43, 44]. We use one scale to assess the practice and content of family psychoeducation (scale 1) and a general organisational index (GOI) scale (scale 2) to assess the organisation, penetration rate, and general integration of family psychoeducation in the unit’s clinical practice. These scales were used in BPB and demonstrated robust psychometric properties [45, 46]. The third scale (scale 3) gives a composite assessment of structure, content, implementation, and penetration rate of basic family involvement and support. The project group developed the latter scale to measure other elements of the IFIP intervention. Thus, our fidelity instruments measure both fidelity and penetration rate, as defined by Proctor et al. [42].

#### Data collection

Project members will measure fidelity on site visits, by the aid of interviews with clinicians, leaders and resource-persons, as well as written material, observations, and quantitative data (e.g. the number of eligible patients who receive family psychoeducation). Each assessment team will consist of two persons to counteract bias and be able to calculate inter-rater reliability. The raters will score fidelity independently and then sort out any discrepancies to reach a consensus score. Clusters in the intervention arm will be scored at baseline, and with new assessments at 6, 12, and 18 months after the implementation start date, whereas units in the control arm will be measured at baseline and 18 months only. This is both to allocate our resources effectively and to avoid influencing the control clusters through repeated fidelity measurements.

#### Outcomes and data analysis

Project members scored baseline fidelity before randomisation of the clusters, to counteract experimenter bias. To complete objective three, we will examine the baseline fidelity scores and analyse their distribution in both arms, while exploring contributing factors such as cluster characteristics. We will investigate the psychometric properties of all three fidelity scales. When addressing objective two, we will compare change in fidelity to the intervention after 18 months, between the two arms, controlling for baseline fidelity and other relevant covariates. These latter changes constitute the IFIP trial’s only primary outcomes. These outcomes will be reported as change in total fidelity, change in fidelity scales 1, 2, and 3 separately, and for scale 3; change in the subscale for penetration rate and change in the subscale for content, structure and implementation, respectively. The two additional fidelity measurements in the intervention arm will help us monitor and influence the implementation process closely. We will employ analysis of variance (ANOVA) models for the statistical analysis.

### Patients’ and relatives’ quantitative outcomes

The main purpose of this part is to determine whether a higher level of implementation of family involvement is associated with relevant and favorable outcomes for patients and relatives, as put forth in objective six.

#### Sample size

All the outcomes of this part are secondary outcomes. With regards to sample size however, for patients we elected the ‘interpersonal relationships’-subscale from the Behavior and Symptom Identification Scale (Basis-24) (questions 4–8). This instrument covers six domains: depression/ functioning, interpersonal relationships, self-harm, emotional lability, psychosis, and substance abuse, as seen from the patient’s perspective, and has shown good reliability and validity [47]. For relatives we chose the outcome ‘experienced support’ measured with the Carer Well-being and Support (CWS) questionnaire short version 2 part B. This part measures support from the health services, as experienced by the relative, with demonstrated good reliability. However, validity for this scale was not available, due to the lack of appropriate validating measures [48]. Since we have not found comparable studies that have published data on these instruments, we decided to use a 0.5 SD improvement (medium effect) when calculating the sample size. With 80% power and 5% two-tailed significance, we would need 64 patients and 64 relatives in each arm, in a study with individual randomisation. For our cluster randomised trial, assuming an intraclass correlation coefficient (ICC) of 0.05 and having 7 clusters in each arm, we need 112 patients and 112 relatives in each arm. Calculation is done as defined for cluster randomised trials in health services research [49, 50] and the elaboration of the CONSORT statement in relation to cluster randomised trials [51, 52]. Taking into account the possibility of a 30% drop out, we need to recruit 161 patients and 161 relatives per arm.

#### Recruitment

Local clinicians will assess the patients in their respective unit for eligibility and competence to consent to participate. If a patient fulfills the criteria, the clinician informs the patient about the study and, if he or she wishes to participate, obtains a written informed consent. The clinician will then ask for permission to contact the closest relative to inform and possibly include her or him. We would like to include patients and relatives in dyadic pairs, but if this proves difficult, we might include relatives and patients separately. The recruitment process will follow a written and uniform procedure in both arms, where every eligible patient is asked to participate, to counteract selection bias. The inclusion period will start 1 month before the implementation start date, and continue for 12 months. Since many patients are discharged from specialist health care services to follow-up in their local municipalities after 1–2 years, recruitment has to start after the randomisation of clusters. This is to ensure that participating patients are still in treatment when the intervention units are ready to begin implementation. The clinical units will receive financial compensation for each pair recruited, and to promote retention, patients and relatives will each receive a symbolic compensation (gift card) after completing the third questionnaire. When this manuscript was submitted, the trial was actively recruiting patients and relatives.

#### Data collection and outcomes

Clinicians will fill in a questionnaire with the patient’s demographic, social, and clinical data at inclusion. They will also score the Global assessment of functioning scale (GAF), split versions for symptoms and functioning [53], along with the Health of the Nation Outcome Scale (HoNOS). The latter instrument contains 12 items on a 5-point Likert-scale, assessing clinical problems and social functioning with reasonable adequacy. HoNOS has been generally acceptable to clinicians who have used it, is sensitive to change or the lack of it, showed good reliability in independent trials and compared reasonably well with equivalent items in the Brief Psychiatric Rating Scales and Role Functioning Scales [54]. Both instruments will be repeated after 12 months or upon discharge. Before starting recruitment, clinicians attended a 1.5-h long course in scoring HoNOS and GAF, to improve the reliability of these measurements. In the majority of our clinical sites, GAF was in frequent use and HoNOS was familiar to some clinicians. We choose these instruments partly because of their brevity to reduce the burden on local clinicians.

At inclusion only, relatives will provide general demographic and social data about themselves, and patients will be screened for drug and alcohol abuse with the 11-item ‘drug use disorders identification test (DUDIT) and the 10-item ‘alcohol use disorder identification test’ (AUDIT), respectively. Both self-reported instruments have shown satisfactory psychometric properties in clinical and non-clinical samples [55, 56].

Patients and relatives will fill in their respective questionnaires at inclusion, 6, and 12 months, containing the self-reported variables and instruments in Table 1. The self-reported instruments assess the psychosocial health of patient and relative, their experience of the mental health services, including shared decision-making, and the emotional climate between patient and relative. The latter is a primary target for family psychoeducation, whereas the two first domains might be affected by various degrees of improved family involvement and support. At the same time points, exposure to family psychoeducation for patients and relatives, and exposure to involvement and support measures for relatives will be reported. Adherence with medication will be monitored with a single question to the patient, relative and clinician.

**Table 1:** Self-reported variables and instruments at inclusion, 6 and 12 months

Variable	Instrument	Items	Scale^a^
**Patients’ self-reported outcomes**			
Experience of mental health and functioning	**Basis-24** [47], The Behavior and Symptom Identification scale	24	L-5
Quality of life	**ReQoL-10** [57], The Recovering Quality of Life questionnaire	10	L-5
Perceived criticism and warmth from relative	**PCW** [58], Perceived criticism and warmth	5	L-10
Experienced shared decision making	**CollaboRATE** [59]	3	L-10
General satisfaction	**MANSA** [60], the Manchester Short Assessment of Quality of Life – first item	1	L-7
Experienced burden of mental health problems	IFIP trial question	1	L-7
**Relatives’ self-reported outcomes**			
Experienced support	**CWS v2** [48], Carer Well-being and Support questionnaire, short version part B	18	L-4
Experience of caregiving	**ECI** [61], The Experience of Care-giving inventory questionnaire	66	L-5
Caregiver quality of life	**CarerQoL** [62], The Care Related Quality of Life questionnaire	7	L-3
Experienced shared decision making	An instrument inspired by **CollaboRATE** [59]	3	L-10
Expressed emotion	**FQ** [63], The Family questionnaire	20	L-4

^a^ *L* Likert scale and number of steps for each item

Number of psychiatric hospital admissions and days spent in hospital for patients will be obtained from national registries, for the period of 18 months before and 18 months after inclusion. Use of public health resources and work participation will be recorded for both patients and relatives over the same period of time, with data from national registries.

#### Data analysis

All analyses will be conducted on an intention-to-treat (ITT) basis. The primary analysis will be carried out by the use of generalised linear mixed models (GLMM), to test differences in outcome measures for patients and relatives between the intervention and control groups, as well as moderator effects. To investigate possible mediating factors, we will use techniques from modern causal mediation analyses. In order to take into account the trial design in which patients and relatives (level 1) are nested within treatment units (level 2), the treatment units will be included in the models as a random effect in accordance with CONSORT guidelines for cluster randomised trials [51]. Multiple imputation procedures will be used to manage missing values of individual characteristics. To assess the robustness of the findings, tests will be redone by only including the subset of patients/relatives with complete outcome data at 6 and 12 months. Tests will also be redone by only including the subset of patients/relatives who still satisfy the inclusion criteria (F20–29 diagnosis) at 12 months. To address objective six, we will investigate whether higher fidelity scores are associated with improved outcomes for patients and relatives, within the same model setup as described above.

### Blinding

For obvious reasons, local clinicians and project members providing the implementation support cannot be blinded to the clinical units’ allocation status. The project’s researchers also contribute to the implementation program, and will accordingly neither be blinded. However, most of the data gathered by project members is either self-reported or retrieved from national registries, and therefore less susceptible to experimenter bias. Patients and relatives will not be informed about their clinical unit’s allocation status until after they have agreed to participate, to counteract selection bias.

### Qualitative outcomes

In this part, we seek to explore the implementation process, including barriers, facilitators, ethical dilemmas, conflicting interests and other aspects, both positive and negative, of family involvement during psychotic disorders, from multiple perspectives to address objectives 4 and 5. In addition, we will employ qualitative data to assist the implementation process directly, by identifying and dealing with barriers and ethical dilemmas.

We will conduct semi-structured interviews with members of each respective stakeholder group (patients, relatives, and clinicians), during the middle of the implementation period. For relatives, we will have 3–6 focus groups with 3–8 participants each and a similar number for clinicians, with the possibility to conduct individual or additional interviews with the same group when necessary. About 10–15 patients will be interviewed individually, with the option of having focus groups where feasible. We will only include patients, relatives and clinicians from the intervention arm and the sampling will be purposive in the sense that we wish to have participants with different experiences of, and views on, family involvement. Relatives and patients can be recruited both through the local clinicians and from the participant pool in the patient- and relative study. We will explore the stakeholders’ perspectives on current family involvement practices in their unit, the selected recommendations, barriers and facilitators, ethical dilemmas, and positive and negative experiences with family involvement and with the implementation project. The unit’s implementation team (3–8 members) will form separate focus groups, one per intervention cluster, at the beginning of the implementation period and in the middle of it. These interviews will cover the same issues, but place particular emphasis on barriers, facilitators, ethical dilemmas, and the implementation process. All interviews will be recorded digitally, and written consent will be obtained from the participants.

Project members will transcribe the interviews verbatim and the main analytic strategy will be manifest qualitative content analysis, using the topics in the interview guide as a starting point for the analysis, and inspired by relevant theories from the fields of ethics, implementation- and social science. However, the analysis will also allow for emerging and latent themes through a more naïve reading of the transcribed text. In addition, the project group will seek to integrate other ethnographic kinds of qualitative data, such as field notes and document analysis to obtain a more comprehensive understanding of the implementation process, the institutional context, and the research questions.

### Health economics

To meet objective seven, we will evaluate whether improved outcomes for patients and relatives are justified by the costs of implementing family involvement for persons with psychotic disorders, in a cost-effectiveness analysis. Based on this analysis, we aim to create a realistic overview of implementation costs and address possible risks associated with scaling up, such as austerity.

First, we will assess the nature and extent of costs and resources needed to enable and support family involvement. All intervention sites will be asked to register data about the costs of implementing family involvement, and further to add the implementation-related costs covered by our project. To compare the cost of systematic implementation of family involvement to ‘implementation as usual’ (see discussion), we will collect various baseline economic data from the clinical units, such as annual budgets, range of services, direct, indirect and investment costs of current family work, to identify the average cost of different therapeutic sessions. Variation in cost levels between centres will be accounted for by assigning a distribution to the average cost.

Second, we will perform a cost-effectiveness analysis, by comparing the costs and health outcomes for patients and relatives. The health outcome will be estimated by quality adjusted life years (QALYs), calculated from CarerQol-7D for relatives and ReQoL-10 for patients. Costs will be estimated from both a health care- and societal perspective. Health care utilisation for patients and relatives, such as hospital admissions, appointments with different health providers, length of stay/number of treatments, day care and medication use will be included in the health care perspective. In the societal perspective, informal care (caregivers time allocated to care), and production loss due to absence from work for patients and relatives will be included. Production loss among those not in the work force (unemployed, retired and at home) will be discussed.

Statistical analysis will consist of estimating the total costs and health outcomes of both systematic implementation and ‘implementation as usual’. The results will be presented by the incremental cost-effectiveness ratio (ICER), defined by the incremental costs (differences in cost of systematic implementation versus ‘implementation as usual’) to the incremental QALYs (differences in total QALYs of systematic implementation versus ‘implementation as usual’). Uncertainty will be displayed by the bootstrap method, a non-parametric approach. Based on the cost-effectiveness analysis a budget impact analysis of scaling up the intervention will be estimated.

### Political economy analysis

This part of the study will explore facilitators for and barriers to successful implementation of family involvement on a broader sociocultural, institutional and political level by the use of political economy analysis (PEA), thereby addressing objective four [64, 65]. PEA is concerned with the interaction of political and economic processes in a society such as interests and initiatives, the role of the formal institutions (e.g. legislation and policy making), structural aspects, the impact of norms, values and ideas, and the distribution of power and wealth between different groups and individuals, and the processes that create, sustain and transform these relationships over time [66]. Subsequently, PEA situates the implementation strategies of family involvement in a broader understanding of the prevailing political and economic processes [67] and is useful to increase dialogue and reduce conflicts amongst stakeholders and to provide more effective policy and political programs on the targeted issue.

A document analysis will be performed on a sample of previous research, selected official publications and country-wide surveys from the period of about 2000–2018 concerning the most important relevant historical and current policy development, legal framework, health economy aspects and educational programs and codes of ethics for key professions that might influence stakeholders’ perceptions and affect the implementation of the national guidelines. The sample is based on a combination of desk research with search on relevant literature bases/websites, snowballing/reference nesting, and information from key experts.

Moreover, semi-structured focus group interviews with a purposive sample of key stakeholders from: a) politicians on a national level (*n* = 1), b) national health authorities (n = 1), c) national organisations dealing with complaint cases in mental health care (n = 1), d) professional associations and service user- /next-of-kin- organisations (*n* = 3), e) the regional health trust administration (n = 1), f) local health trust administration (*n* = 2–3) and g) political/administrative stakeholders from municipalities (n = 2–3), amounting to 11–15 interviews in total. We aim for larger focus groups with up to 10–15 participants, since this might give us a broader picture of considerations and contribute to display influencing power relations, interests and incentives through group interactions. However, the interview design will be flexible and adjusted to the preferences of informants (e.g. individual interviews) to ensure sufficient participation and information. Semi-structured interview guides, developed and adjusted to each stakeholder group, will address political/policy-making, legal and financial issues as described above, as well as interests, power relations and structural and cultural/ideological incentives regarding family involvement. Written consent will be obtained before the interviews, and the interviews will be recorded digitally and transcribed verbatim.

The main objective of the analysis is to identify barriers and facilitators on a political and institutional level that might be addressed to improve implementation of family involvement in CMHCs, by drawing on the analytic framework for PEA as developed by the Department for International Development (DFID) [64, 65]. The document analysis will make use of a combination of discourse analysis and content analysis, while the interviews will undergo a qualitative thematic content analysis [68–70]. The interview analysis and document analysis will be integrated with other data sources from the trial in the overall PEA, including future policy assessments before publication. Final choice of data analysis and assessments strategies will be decided upon after a closer consideration of the collected data material.

### Data management and monitoring

All collected data will be stored in the University of Oslo’s secure database (In Norwegian ‘Tjenester for Sensitive Data’ - TSD) and only project members will have access to the storage area. Questionnaires filled in online in the University’s ‘nettskjema’-application will be encrypted and stored directly in TSD. Questionnaires filled in on paper will be stored securely at the clinical units, before a project member transfers them to TSD. Personal data will always be stored separately from questionnaires, in the form of a code list/encryption key. A local research coordinator at each clinical unit will supervise local data collection and storage. The University of Oslo has signed individual contracts with each participating Health Trust, which specifies responsibilities for data collection and storage in accordance with Norwegian legislation. Since IFIP is a minimal risk trial, we do not have a data monitoring committee, but project members monitor the recruitment process and collection of outcomes closely to ensure conformity with the trial’s ethical and methodological standards. Each study site has at least one designated project member to oversee and assist the implementation process (in the intervention arm) and data collection (in both arms).

### Research ethics

The study will only include participants who are competent to make the decision to participate in research. We will obtain both oral and written consent and the participants can withdraw from the study at any time, without giving any reason and without experiencing any consequences for their treatment. Patients with psychotic disorders can be considered a particularly vulnerable group and we have made considerable efforts to make our research responsive to their needs, while also ensuring that they stand to benefit from the knowledge we may generate.

By using a cluster randomised design, the project needs to include more patients than would a study with individual randomisation, and it is therefore important that the choice of study design is justified. Consent to be exposed to our intervention is also sought at cluster level and not from patients and relatives within the cluster. Family involvement is a low-risk intervention and the local clinicians must assess whether it is contraindicated for certain patients and relatives. We also maintain that the treatment options in the intervention clusters will improve, and that we do not reduce the quality of the services offered in the control arm. After the implementation period, we will offer training and guidance to the control clusters as well.

In the political economy analysis we will interview political leaders and health administrators about issues which might be controversial. Therefore, confidentiality and possibilities for additional individual interviews will be underlined. The interviews will not gather information in terms of personal or political party nature, and the results will have to be published in a generalised way, without reference to their particular source.

All our procedures are in accordance with national and international standards for research ethics, including the Helsinki Declaration [71]. The study has been approved by the Norwegian regional committee for medical and health research ethics (REC) South East with registration number 2018/128. Important protocol modifications will be reported to REC, and the trial registry at clinicaltrials.gov will also be updated.

## Discussion

The cluster randomised design of the IFIP trial will help us compare implementation-, service- and client outcomes between intervention and control arm. We will not compare the effectiveness of different implementation strategies or the effectiveness of different family interventions. Rather, we seek to combine recommended clinical interventions with recommended implementation interventions and compare the results of their systematic implementation with ‘implementation as usual’ [72]. We use the term ‘implementation as usual’ rather than ‘treatment as usual’ because the project will not prevent the control clusters from improving their family involvement practices, and there are considerable incentives for them to do so. The implementation of new clinical pathways in Norwegian mental health services coincides with the implementation period of our study. These clinical pathways set standards and deadlines for documentation, diagnostic evaluations and treatments, including family involvement practices, and will probably affect both intervention and control conditions. Since we measured baseline fidelity before the clinical pathways were launched, we might be able to monitor some of these effects.

Another challenge for our study is the timing of the inclusion of relatives and patients. To avoid selection bias, it would be optimal to include them prior to the randomisation of clusters. However since the implementation of complex practices requires time, we would risk that many patients would be discharged before being exposed to the intervention. By recruiting patients and relatives in the early- and mid-phases of the implementation period, they are likely to have various degrees of exposure to family involvement practices at inclusion. We hope to monitor parts of this exposure through the questionnaires.

Our trial may contribute to the paradigmatic change in mental health services towards working with relatives, building on the scientific evidence and moral arguments in favour of a family-oriented treatment approach. This study will employ a whole-ward strategy to implement family involvement for persons with psychotic disorders to make it an integrated part of every clinician’s practice, rather than the domain of especially motivated personnel. Through our implementation support, we seek to alter both clinical practice and the structural and organisational conditions that may sustain this effort over time. This requires family involvement to be embedded in daily clinical activities, through routines, checklists and documentation [22]. At the same time, we recognise that many of the barriers to implementation represent genuine ethical dilemmas and conflicts of interests.

We also use a whole-ward research strategy, in the sense that we use broad inclusion criteria and do not require exposure to any specific intervention for our participants. In addition to the whole-ward strategy, our study has several characteristics that combined, to our knowledge, constitute a novel approach. We seek to implement a basic level of family involvement and support and family psychoeducation at the same time. Our study has a strong focus on sustainability and feasibility, where we encourage the clinical units to integrate some implementation interventions as part of their permanent structure. We employ responsive evaluation to ensure that both implementation and research is responsive to the needs of clinicians, patients and relatives. The IFIP trial includes patients and relatives in dyadic pairs and measures outcomes on multiple levels with both qualitative and quantitative methods. Finally, we also aim to see family involvement practices in a broader societal and public health context, by conducting a cost-effectiveness analysis and a political economy analysis. Our study may provide valuable knowledge to the fields of family involvement, mental health service research and implementation science.

## Supplementary information

**Additional file 1.** The IFIP Intervention. Detailed description of the trial’s intervention.

## Data Availability

Not applicable.

## Abbreviations

ANOVA: Analysis of variance
BPB: Bedre psykosebehandling
CFIR: Consolidated Framework for Implementation Research
CMHC: Community Mental Health Centres
DFID: Department for International Development
DPS: Distriktspsykiatrisk Senter
FACT: Flexible Assertive Community Treatment
GLMM: Generalised linear mixed models
GOI: General organisational index
IFIP: Trial acronym: Implementation of Family Involvement for persons with Psychotic disorders
ICC: Intraclass correlation coefficient
ICER: Incremental cost-effectiveness ratio
ITT: Intention-to-treat
MRC: Medical Research Council
NEBP: National Evidence-Based Practices project
QALY: Quality adjusted life years
PEA: Political economy analysis
REC: Regional committee for medical and health research ethics
SD: Standard deviation
TSD: Tjenester for Sensitive Data
