# Supplementary material for: Implementation of guidelines on family involvement for persons with psychotic disorders in community mental health centres (IFIP): protocol for a cluster randomised controlled trial
Source: BMC Health Serv Res. 2020 Oct 9;20:934. doi: 10.1186/s12913-020-05792-4 (PMC7547488; doi:10.1186/s12913-020-05792-4)
Supplement: Supplementary file 1 — Additional file 1. The IFIP Intervention. Detailed description of the trial’s intervention. [file 12913_2020_5792_MOESM1_ESM.pdf]

The IFIP intervention

(The practices and structures marked with an asterisk (*) are recommended in the national guidelines and therefore intended to continue after the IFIP trial is finished).

1. Clinical interventions

    1.1 A basic level of family involvement and support
    1.2 Family psychoeducation in single-family groups

2. Implementation interventions

    2.1 Training and guidance of health care personnel
    2.2 A family coordinator
    2.3 Other implementation measures

1. Clinical interventions*

1.1 A basic level of family involvement and support

1.1.1 Basic assessment, structure and documentation

Clinicians should make sure:

- That relatives/next of kin are identified and documented. This includes documenting children, younger siblings and the extended network.
- That the patient is invited to a meeting dedicated to discuss family involvement, consent to family psychoeducation and other relevant issues. As a rule, this should take place before meetings with the relatives.
- That adult relatives are invited to a meeting dedicated to discuss family involvement, consent to family psychoeducation and other relevant issues. In general, this should take place without the patient.
- That relatives and the patient are invited to at least one meeting together with health care personnel.
- That the patientʹs primary clinician attends at least one of the meetings with the patient and relatives together.
- That a crisis plan is developed, regularly updated and that relatives have contributed to, or at least been made familiar with, its contents.
- That family involvement, psychoeducation and the crisis plan are documented in the patientʹs discharge report.

1.1.2 At least three meetings dedicated to family involvement and support

Meeting with the patient:

A meeting with the patient, without the relative(s) present, dedicated to discuss family involvement, consent to family psychoeducation, and other relevant issues. The meeting should be guided by a written checklist, documented in the patient records and cover the following items:

- Ask the patient directly: ʹWhat is important for you to discuss with regards to family involvement?ʹ Then, make sure the conversation covers these topics.
- Ask the patient about his/her relationship to the relatives, including any children.
- Investigate whether the patient has experienced violence and/or abuse from the relatives.
- Talk to the patient about family involvement, confidentiality and conflicts of interest (how and why). Talk to the patient about his/her rights, and the different roles and responsibilities of patient, relatives and health care personnel. Make sure to elicit the patientʹs concerns and preferences.
- Talk about issues connected to having younger children, their needs and parent responsibilities, if relevant.
- Systematically recruit patients with primary psychotic disorders to participate in psychoeducation in single-family groups. If the patient refuses, one should try to uncover why. If not contraindicated, one should ask again later, and consider this a continuous process. This applies to basic family involvement outside the psychoeducative model as well.

Meeting with the relative(s):

A meeting with the relative(s), usually without the patient, dedicated to discuss family involvement, consent to family psychoeducation, and other relevant issues. The meeting should be guided by a written checklist, documented in the patient records and cover the following items:

- Ask the relative directly: ʹWhat is important for you to discuss with regards to family involvement?ʹ Then, make sure the conversation covers these topics.
- Talk to the relative about roles, responsibilities and regulations concerning family involvement, confidentiality and documentation.
- Ask how the relative experience his/her relationship to the patient. Listen to the relativeʹs concerns and receive his/her information about the patient.
- Identify the tasks, resources, carer burdens and strengths of the relative to assess his/her need for support, and give him/her advice on where to obtain it.
- Talk to the relative about common economic, social and health related issues connected with being a carer. Talk about strategies on how to handle these issues and where to obtain further support, if needed.
- Where relevant, talk about having younger children, about the parental role and responsibilities and what information and follow-up the children need, and have received.
- Investigate whether the relative(s) have experienced violence and/or abuse from the patient.
- Systematically recruit relatives of patients with primary psychotic disorders to participate in psychoeducation in single-family groups. If the relative refuses, one should try to uncover why. If not contraindicated, one should ask again later, and consider this a continuous process. This applies to basic family involvement outside the psychoeducative model as well.

Meeting together with health care personnel:

This might be a short introductory meeting, before the separate meetings, to agree on what issues can be discussed there. It might also be a longer meeting, to sum up what can be shared from the separate meetings. The separate and joint meetings might form the initial phase of family psychoeducation, or not, depending on the patientʹs and the relativeʹs consent. In any case, the separate meetings should cover the items listed in the two previous sections.

1.1.3 Information to relatives and patients

The clinical unit should:

- Have written information about the unitʹs family work available (how and why), and routinely distribute it to patients and relatives. It should also include information on relevant web resources and support groups.
- Have an overview of local units, organisations, agencies and people who could offer support within or outside the health services, and make sure this information reaches the relatives.
- Arrange seminars/information meetings for relatives on pertinent topics, at least two times a year.

1.2 Family psychoeducation in single-family groups

Family psychoeducation is a structured family intervention, based on the works of Falloon, Boyd and McGill (1) and Anderson, Reiss and Hogarty (2), and consists of the following elements:

- Initial sessions with the patient and relative(s), where the intervention is presented.
- Separate alliance sessions with patient and relative(s) with:
  - Mapping of warning signals.
  - Development of a crisis plan.
  - Mapping of the extended network of the patient.
  - Establishing goals for the treatment.
- Teaching sessions with the patient and relative(s) together. Should consist of the following themes:
  - Understanding and discussion of symptoms.
  - Cognitive difficulties and how they affect activities of daily living.
  - The stress/vulnerability model. Understanding and mapping of different stressors.
  - Coping strategies and family support.
- Communication skills and exercises.
- Problem-solving sessions. Practical and structured solving of problems related to the patient's illness.
- If the patient or the relative(s) will not consent to participate in sessions together, the family workers should offer to perform separate sessions in line with the model.

2. Implementation interventions

2.1 Training and guidance of health care personnel*

2.1.1 Basic education and guidance on family involvement for patients with psychotic disorders

The clinical unit should provide yearly education to all clinical staff on:

- The importance of family involvement and the benefits of following the national guidelines.
- How to approach relatives in a good mannered way and recognise them through small gestures.
- The legal rights and roles of patients and relatives, and the health care services′ obligations towards them.
- How to promote family- and patient involvement during the treatment of primary psychotic disorders, with effective communication and cooperation through the various phases.
- Common challenges related to being a caregiver and where caregivers can obtain support, within or outside the health services. How to provide carers with the relevant information and adequate support.
- Professional, legal and ethical issues one may encounter during family involvement and strategies on how to handle these.

The health care personnel should have:

- Access to guidance in family involvement and support, e.g. from the family coordinator or personnel with expertise in family psychoeducation, from reflection groups or clinical ethics committees.

2.1.2 Training and guidance in family psychoeducation

- All clinical staff and leaders should be offered training and guidance in family psychoeducation sufficient to qualify as group leaders. In our project, certifying training is provided by The Early Intervention in Psychosis Advisory Unit for South East Norway, Oslo University Hospital Trust (TIPS Sør-Øst). It consists of a four-day course and follow-up guidance every sixth week for one year. All participants will be offered a one-day refresher course after one year. The training and guidance in family psychoeducation cover all the elements described in 2.1.1, with emphasis on theory and practical training in alliance sessions, psychoeducation, communication enhancement and problem solving.

2.2 A family coordinator*

- The clinical unit should appoint a designated professional, who receives training and regular guidance, to coordinate family involvement and support.

2.3 Other implementation measures

- The clinical units will constitute a local implementation team of 4-5 persons, working closely with the unit's leader(s) to ensure management commitment and support. The team should include the family coordinator and other central clinicians, and establish systems to gain input and feedback from patients and relatives. The team supervises the local implementation process with assistance from project members.

- The project group will arrange a kick-off session at each intervention unit to provide information and build enthusiasm. It consists of a half-day seminar for all clinical staff, with introductions to family involvement and family psychoeducation, covering some of the elements in 2.1.1.
- The project group will conduct fidelity assessments every sixth month to assess the level of implementation of the intervention.
- The clinical units will receive systematic feedback with each fidelity report, and assistance in setting short- and long-term goals, distributing tasks and making work plans.
- The project group will arrange network meetings where the implementation team, family coordinators and leaders from different units can meet and share experiences, and receive further training.
- The project group will conduct focus group interviews and systematically use the resulting qualitative data to address local barriers, facilitators and ethical dilemmas, to meet objectives four and five. Based on qualitative interviews and other data sources, the project group will develop a comprehensive guide of barriers and ethical dilemmas, with particular emphasis on the potential strategies to overcome these.
- Regular quantitative evaluations of how patients and relatives experience their involvement and support in the clinical unit.*
- The clinical units will share useful tools, written material and examples on good practices with each other through a web page, and in the network meetings. The project group will provide conversation guides/checklists for discussing FI with patients and relatives. We will also provide an overview of important barriers to family involvement and strategies on how to handle them.
- The project group will offer the clinical units the possibility to assess clinicians′ readiness to implement a new practice through the Implementation Process Assessment Tool (IPAT) questionnaire (3). In the relevant units, a strategic sample of up to 15 clinicians will fill in the questionnaire, up to three times during the implementation period. The results will be summarised in a report to the unit′s leader(s), implementation team and clinicians, and can be used actively to guide the implementation process.

1. Falloon IRH, Boyd JL, McGill CW. Family care of schizophrenia : a problem-solving approach to the treatment of mental illness. New York: Guilford; 1984.
2. Anderson CM, Reiss DJ, Hogarty GE. Schizophrenia and the family : a practitioner's guide to psychoeducation and management. New York: Guilford; 1986.
3. Hartveit M, Hovlid E, Nordin MHA, Ovretveit J, Bond GR, Biringer E, et al. Measuring implementation: development of the implementation process assessment tool (IPAT). BMC Health Serv Res. 2019;19(1):721.
